# Emerging roles of tRNA-derived small RNAs in cancer biology

**DOI:** 10.1038/s12276-023-01038-5

**Published:** 2023-07-10

**Authors:** Saebyeol Lee, Jungeun Kim, Paul N. Valdmanis, Hak Kyun Kim

**Affiliations:** 1 Department of Life Science, Chung-Ang University, Seoul, 06974 Republic of Korea; 2 Division of Medical Genetics, Department of Medicine, University of Washington, Seattle, WA 98115 USA

**Keywords:** Small RNAs, Drug development

## Abstract

Transfer RNAs (tRNAs) play an essential role in mRNA translation by delivering amino acids to growing polypeptide chains. Recent data demonstrate that tRNAs can be cleaved by ribonucleases, and the resultant cleavage products, tRNA-derived small RNAs (tsRNAs), have crucial roles in physiological and pathological conditions. They are classified into more than six types according to their size and cleavage positions. Since the initial discovery of the physiological functions of tsRNAs more than a decade ago, accumulating data have demonstrated that tsRNAs play critical roles in gene regulation and tumorigenesis. These tRNA-derived molecules have various regulatory functions at the transcriptional, post-transcriptional, and translational levels. More than a hundred types of modifications are found on tRNAs, affecting the biogenesis, stability, function, and biochemical properties of tsRNA. Both oncogenic and tumor suppressor functions have been reported for tsRNAs, which play important roles in the development and progression of various cancers. Abnormal expression patterns and modification of tsRNAs are associated with various diseases, including cancer and neurological disorders. In this review, we will describe the biogenesis, versatile gene regulation mechanisms, and modification-mediated regulation mechanisms of tsRNA as well as the expression patterns and potential therapeutic roles of tsRNAs in various cancers.

## Small RNAs: potential roles in cancer treatment

A type of small RNA has been shown to be associated with cancer and neurological disorders, and could be used in treatment. Small noncoding RNAs, just a few tens of nucleotides in length, have been referred to as the “dark matter of the genome”, playing unexpected roles in gene regulation. Hak Kyun Kim at Chung-Ang University in Seoul, South Korea, and co-workers reviewed research into tRNA-derived small RNAs (tsRNAs), which are produced when transfer RNA molecules are cleaved by enzymes. More than a hundred types of tsRNA modifications have been identified, some of which are involved in generating tumors while others suppress cancer progression. Abnormal tsRNA expression patterns are closely associated with certain diseases, and could be used as biomarkers in clinical trials. Other tsRNAs could be engineered to combat cancer directly as therapeutics.

## Introduction

The concept that only a small percentage of the DNA enclosed within our genome contains all the essential genetic information in the form of protein-encoding genes can no longer be considered accurate. One of the most striking findings in the last few decades is that over 90% of the genome is transcribed into noncoding RNA (ncRNA) and that these ncRNAs are important regulatory molecules^1^. They have been described as the dark matter of the genome and represent an active area of biomedical research.

The discovery of small interfering RNAs (siRNAs) and microRNAs (miRNAs), which are both 18–25 nucleotides (nt) long, shed light on ncRNA-mediated gene regulation mechanisms. In 1993, the first miRNA to be identified, *lin-4*, was found to negatively regulate *lin-14* expression, controlling the temporal development of *Caenorhabditis elegans (C. elegans)*^2^. At that time, researchers had probably not anticipated the presence of thousands of such regulatory ncRNAs. Later, Craig Mello and Andrew Fire unveiled siRNA-mediated gene silencing mechanisms and the role of siRNAs in cellular protection against viruses and transposable elements^2^. These ncRNAs base-pair with target RNA molecules in a complex with Argonaute (AGO) proteins to induce mRNA cleavage, mRNA degradation, or translational inhibition. This complex of ncRNAs and AGO is referred to as the RNA-induced silencing complex (RISC)^3^. Extensive subsequent research has revealed that miRNAs are involved in various physiological and pathological mechanisms, including cancer development, and they have also been utilized as potential biomarkers and therapeutic targets for cancer treatment^3^.

Another group of small ncRNAs related to cancer and therapeutics comprises tRNA-derived small RNAs (tsRNAs), which are generated from mature or pre-tRNA by ribonuclease-mediated cleavage into 18–40-nt fragments. tRNA molecules (76–108 nt) are essential for protein synthesis. They transfer amino acids to the growing polypeptide chain in ribosomes. While tRNA cleavage products were initially observed in urine samples of cancer patients in the late 1970s^4^, they had been considered nonfunctional molecules for three decades. However, since it was revealed by three groups that tRNA cleavage products could play a role in gene regulation in 2009 and 2010^5–7^, it has become increasingly clear that tsRNAs may be involved in human diseases such as cancers and neuronal disorders.

Due to their size, tsRNAs were initially considered to be a particular form of miRNA. However, their biogenesis differs from that of miRNAs, and their functions are much more versatile, as they play roles in transcriptional regulation, post-transcriptional gene repression, mRNA stability, viral replication, global translation inhibition, translational upregulation, and other regulatory mechanisms^8^. Today, a substantial body of evidence demonstrates that tsRNAs can be potential biomarkers and can be used as therapeutic targets for cancer treatment even though the underlying mechanisms remain elusive.

In this review, we provide an overview of the biogenesis of tsRNAs, their regulatory functions in gene expression, and their expression patterns as well as potential therapeutic applications in various cancers.

## Biogenesis and classification

More than six types of tsRNAs are recognized according to their cleavage positions and size. While more than 10 years have passed since the recognition of tsRNAs as functional molecules, the nomenclature of tsRNAs has not been fully established. To prevent ambiguity and to provide a clear description of individual tsRNA species, we will provide one of the most commonly used names for each molecule with position and length information as well as the initial names used in the original publications.

### Type I 5′ and 3′tsRNAs

**Fig. 1:**
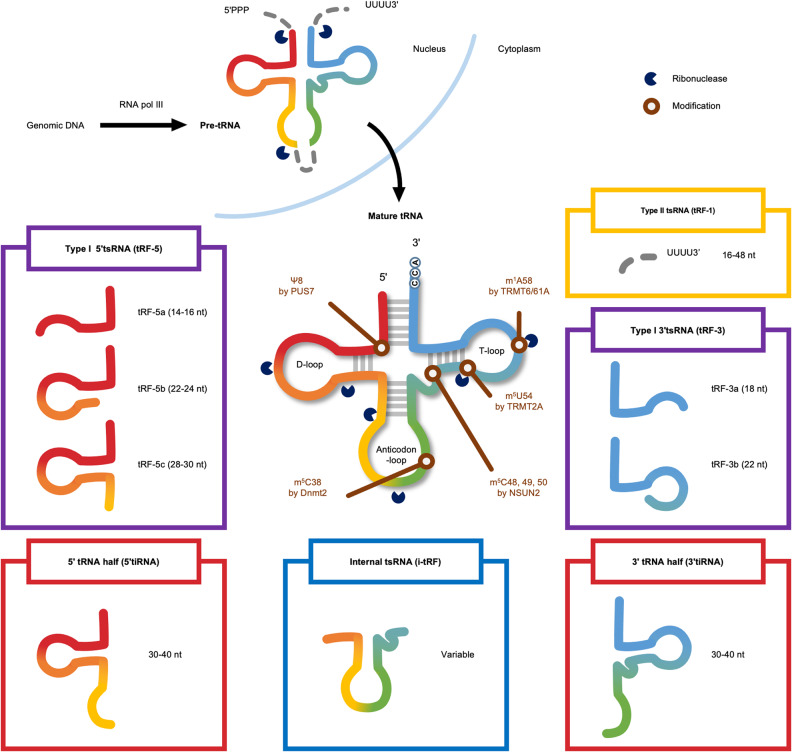
tsRNA classification and modification. tsRNAs are generated from mature tRNAs and pre-tRNAs. They have been classified into more than six types depending on the cleavage site. Each type of tsRNA has a different function and biogenesis mechanism, but the details are unclear. The important modifications and modifying enzymes are shown.

Type I tsRNAs comprise 18–30-nt long tsRNAs derived from mature tRNA molecules and are also called tRNA-derived fragments (tRFs)^5,6^ (Fig. 1) and further divided into groups of 5′tsRNA (tRF-5) and 3′tsRNA (tRF-3). The first group, 5′tsRNAs, includes the region from the 5′-end of tRNA to the D-loop area or anticodon loop. They are also referred to as tRF-5a (14–16 nt), tRF-5b (22–24 nt), and tRF-5c (28–30 nt) molecules according to their length. The second group, 3′tsRNAs, includes the region from around the T loop to the 3′-end of tRNA. They are mostly 18 or 22 nt in length and accordingly named tRF-3a and tRF-3b, respectively. The biogenesis of type I tsRNAs is still not well understood and remains controversial. A number of groups have proposed that Dicer can produce type I tsRNA^9–14^, but there are also other reports indicating that type I tsRNAs are not processed by Dicer^15–17^. These observations suggest that Dicer might process type I tsRNAs from specific tRNAs in particular cell types or under particular conditions. They are also produced by different endonucleases, including Angiogenin and RNase T2, in animals, plants, and yeast, suggesting that tsRNA processing mediated by other ribonucleases needs to be further investigated^15,18^.

A recent study on type I 3′tsRNA biogenesis further demonstrated that tRNA aminoacylation is required for the generation of 22-nt LeuCAG 3′tsRNA which is acylated, suggesting that type I 3′tsRNA might be processed after tRNA maturation and that aminoacylation might have a role in tsRNA biogenesis^19^.

### Type II tsRNAs

Type II tsRNAs (tRF-1) are 16–48 nt in length and are derived from the 3′-trailer sequence of pre-tRNA molecules^5^ (Fig. 1). RNA polymerase III transcribes the pre-tRNA molecule composed of a 5′-leader, a mature tRNA, an intron, and a 3′-trailer sequence (Fig. 1). During tRNA maturation, the 5′-leader and 3′-trailer sequences are removed by RNase P and other RNases, including RNase Z, respectively^20^. Since type II tsRNAs are processed by RNase Z and Elac2, a tRNA 3′-endonuclease and a prostate cancer susceptibility gene^5,6^, they are thought to be generated during tRNA maturation. A number of type II tsRNAs are differentially expressed in various cancers^21–23^. However, their regulation and function need to be further determined.

### Internal tsRNA

Unlike type I and type II tsRNAs, which include either the 3′- or 5′-terminus of parent tRNAs, internal tsRNAs (tRF-2 or i-tRF) straddle the anticodon region of tRNA but do not contain any tRNA termini^24^ (Fig. 1). The biogenesis of internal tsRNAs remains elusive.

### 5′ and 3′tRNA halves (tiRNA)

RNA molecules of 30–40 nt in size produced from tRNAs by cleavage at the anticodon region are called tRNA halves since their length is almost half the size of a mature tRNA (Fig. 1). They are also called tRNA-derived stress-induced RNAs (tiRNAs), as their generation is induced under various stress conditions, such as nutrient starvation, heat shock, hypoxia, oxidative stress, and UV irradiation, in a variety of species, including yeast, *Tetrahymena* species, plants, and mammals^7,25–29^.

Initially, tRNA halves were thought to be processed under stress by angiogenin or Rny1p in mammalian or yeast cells^7,28^. However, a subset of tRNA halves are constitutively expressed in androgen receptor (AR)- or estrogen receptor (ER)-positive cancer cells even though they are processed by angiogenin. Since they are dependent upon sex hormones and their receptors, they are named sex-hormone-dependent tRNA-derived RNAs (SHOT-RNAs)^30^. These observations suggest that tRNA halves can be generated independent of stress, which means that their biogenesis needs to be further investigated.

## tsRNA functional roles

### Transcriptional regulation

The Gullerova group has revealed that tsRNAs produced by Dicer induce gene silencing through nascent RNA silencing (NRS). Type I 18–22-nt 3′tsRNAs and type II tsRNAs produced by Dicer suppress a number of genes associated with various cancers by targeting early introns of nascent RNAs in the nucleus. These tsRNAs can significantly reduce the expression of target genes at both the mRNA and protein levels in a manner dependent on Ago2 endonuclease activity^14^.

### Post-transcriptional regulation

#### AGO-dependent vs. AGO-independent association

**Fig. 2:**
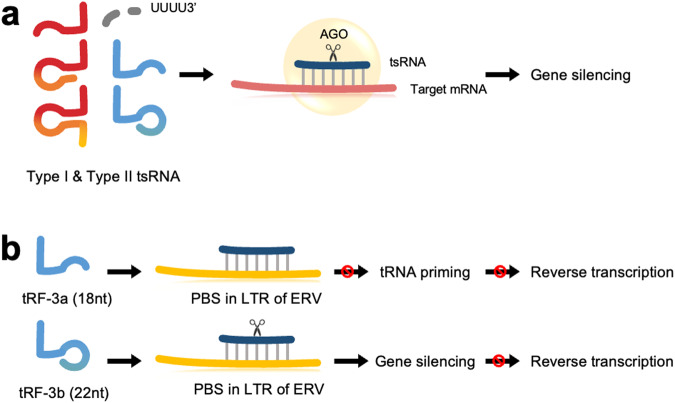
Post-transcriptional regulation. **a** Particular type I or type II tsRNAs bind to target mRNA and induce AGO2-mediated cleavage or mRNA degradation, resulting in gene silencing. **b** tRF-3a binds to the primer binding site (PBS) in the long terminal repeats (LTR) of endogenous retrovirus (ERV), blocks tRNA priming and inhibits reverse transcription. tRF-3b binds to PBS in the LTR of ERV, induces gene silencing and inhibits reverse transcription.

In the early phases of tsRNA research, it was suggested that tsRNAs might have gene silencing ability similar to miRNAs based on the association of tsRNAs with Argonaute (AGO) proteins and their size, which is similar to that of miRNAs (Fig. 2a).

Initially, the Kay group demonstrated that several type I and type II tsRNAs interact with overexpressed AGO3 and AGO4 more than they interact with AGO1 and AGO2 according to immunoprecipitation (IP) experiments^6^. Then, the John group identified that 17-nt-HisGTG3′tsRNA and 18-nt-LeuCAG3′tsRNA are associated with endogenous AGO2 by northern blotting and IP experiments and also revealed that these tsRNAs induce AGO2-mediated cleavage of artificial target mRNAs. Their findings suggest that HisGTG and LeuCAG 3′tsRNA might regulate gene expression in a manner similar to miRNA-mediated regulation^15^. In addition, the Dalla-Favera group—by performing IP experiments—assessed whether CU1276 tsRNA (GlyGCC3′tsRNA) interacts with overexpressed AGO1–4 proteins^11^. By analyzing published photoactivatable-ribonucleoside-enhanced crosslinking and immunoprecipitation (PAR-CLIP) and crosslinking, ligation, and sequencing of hybrids (CLASH) data, the Dutta group also found that type I tsRNAs are more associated with AGO1, AGO3, and AGO4 than with AGO2, while type II tsRNAs barely interact with the AGO proteins^16^. The association of tsRNAs and AGO proteins has also been observed in *Drosophila*, where the interaction of tsRNAs with AGO1 and AGO2 changes with aging^31^.

A limited number of biological targets of Ago-associated tsRNAs have been identified, and a growing body of evidence has demonstrated that tsRNAs might not be associated with AGOs and have functions that differ from those of miRNAs. The Hutvagner group performed size fractionation of HeLa S100 extract and IP experiments with human AGOs and suggested that small RNAs derived from tRNA^Gln^ have no significant interaction with AGOs^10^. The Kay group also confirmed the lack of interaction between tsRNA and AGOs by demonstrating that several tsRNAs are not associated with endogenous AGO proteins^32^. The Croce group reported that ts-4521 (SerGCT type II tsRNA) and ts-3676 (ThrAGT type II tsRNA and ThrCGT type II tsRNA) levels are downregulated, and they are mutated (~2%) in chronic lymphocytic leukemia (CLL), the most common form of human leukemia. Interestingly, these tsRNAs interact with Piwi-Like protein 2 (PIWL2) instead of AGO proteins^21^. The Gunter group found that the Lupus autoantigen (La) protein prevents tsRNAs from interacting with AGO proteins^33^. La is a single-stranded RNA-binding protein that binds to the UUU stretch at the 3′-end of Pol III transcripts, including tRNA, and protects them from exonuclease activity, ensuring the correct folding of the RNA^33^. La interferes with the production of tRNA fragments and the formation of tsRNA and the AGO1–4 protein complex^33^.

All these reports suggest the possibility that tsRNAs might act as miRNAs, but many of them exhibit weak interactions with AGO proteins. Therefore, they might have different biological functions than miRNAs.

#### Regulation of viral replication

The Bao group delineated the regulatory role of tRNA halves in respiratory syncytial virus (RSV) replication. RSV infection induces the formation of a subset of tRNA halves, such as tRF5-GlyCTC, tRF5-GlyGCC, tRF5-LysCTT, and tRF5-CysGCA^34,35^, which are present in the cytoplasm and have transgene silencing activity. However, the mechanism of this suppressive activity might be different from that of miRNAs, since their seed sequences do not comprise the first 2–7 nt, unlike miRNA^34,35^. RSV infection-induced tRF5-GluCTC promotes RSV replication by repressing apolipoprotein E receptor 2 (APOER2), an antiviral protein that inhibits RSV replication^36^. Human T-cell leukemia virus type 1 (HTLV-1) is etiologically associated with adult T-cell leukemia (ATL). The D’Agostino group found that tRF-3019 (18-nt Pro 3′tsRNA) is expressed upon HTLV-1 infection and binds to the primer binding site (PBS) of HTLV-1 and acts as a primer for reverse transcription^37^. These results suggest that the group of tsRNAs involved in viral replication needs to be further delineated to facilitate cancer treatment since both tsRNAs are known to be highly oncogenic viruses.

#### Regulation of transposon element (TE)

The translocation activity of TEs is detrimental to the host genome. Thus, TE transcription is usually suppressed through epigenetic modifications^38^. The Martienssen group discovered that the 18-nt 3′CCA tRF (18-nt 3′tsRNA) and 22-nt 3′CCA tRF (22-nt 3′tsRNA) sequences are complementary to the tRNA primer binding site (PBS), which is essential for endogenous retrovirus (ERV) reverse transcription, in long terminal repeats (LTR) of ERVs. The 18-nt 3′CCA tRF binds to the PBS of the ERVs and blocks tRNA priming, which inhibits reverse transcription (Fig. 2b). However, a 22-nt 3′CCA tRF inhibits reverse transcription by inducing post-transcriptional silencing^39^ (Fig. 2b).

### Translational regulation

#### Global inhibition

Under stress conditions, tsRNAs can contribute to translational inhibition to minimize protein synthesis. The levels of tRNA halves are elevated under stress, as mentioned in the previous section. In general, cancer cells are exposed to various forms of stress, including oxidative stress, glucose deprivation, and hypoxia. Thus, increased levels of tRNA halves under stress conditions may be associated with cancer progression.

Cells inhibit global protein synthesis under stress to conserve energy for repairing stress-induced damage through two different pathways. The first pathway is dependent on the phosphorylation of eukaryotic translation initiation factor 2α (eIF2α) and stress granule (SG) formation^40–42^. The other pathway is independent of eIF2α phosphorylation^43^.

**Fig. 3:**
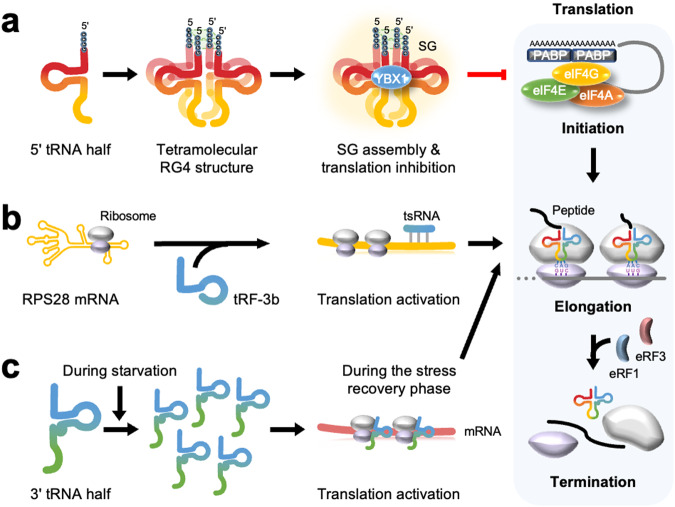
Translational regulation. **a** Global inhibition. Angiogenin-induced 5′tRNA half with a terminal oligoguanine (TOG) motif inhibits translation in a phospho-eIF2α-independent manner. The 5′-TOG motif folds into tetramolecular RNA G-quadruplex (RG4) structures and induces stress granule (SG) assembly, which leads to translation initiation inhibition. Y-box binding protein 1 (YB-1 or YBX1) is required for SG assembly. **b** Translational activation. LeuCAG 3′tsRNA binds to RPS28 mRNA and alters the secondary structure, leading to the activation of translation. **c** Translational activation in *Trypanosoma brucei* (*T. brucei*). The production of Thr 3′tRNA half is promoted during starvation and binds to ribosomes during the stress recovery phase to activate translation.

The Anderson group, investigating the mechanism of tsRNA-dependent global translation inhibition, found that angiogenin-induced 5′-tiRNA generation inhibits global translation by phospho-eIF2α-independent SG assembly^7,44^ (Fig. 3a). They further reported that a terminal oligoguanine (TOG) motif is required for tiRNA-induced translational repression^45^. The only human tRNAs with TOG motifs (4–5 guanine residues) at the 5′ end are tRNA^Ala^ and tRNA^Cys^. The 5′-TOG motif folds into G-quadruplex (G4)-like structures and replaces eukaryotic initiation factor 4 F (eIF4F), thereby inducing SG assembly and translation inhibition^46^. G4s are guanine-rich nucleic acid structures composed of G-quartet motifs where four guanine molecules interact with each other through hydrogen bonding. RNA G4 (RG4) structures are also involved in several biological processes, and thousands of intramolecular RG4s have recently been identified in the human transcriptome. According to most reports, RG4s in mRNA inhibit translation by acting as a roadblock for scanning preinitiation/ribosomal complexes or by attracting translation inhibition factors^47^. The tetramolecular RG4 structure of the 5′-TOG motif interacts with the HEAT-1 (huntingtin, elongation factor 3, protein phosphatase 2A, and target of rapamycin [TOR]) domain of eukaryotic translation initiation factor 4G (eIF4G), a component of the eIF4F complex, disrupting the stability of the eIF4F complex. Targeting eIF4G impairs 40S ribosome scanning on mRNA, leading to the formation of eIF2α-independent SGs^48^.

Multifunctional cold-shock domain (CSD)-containing Y-box binding protein 1 (YB-1 or YBX1) has been identified as a component of 5′-TOG-tiRNA ribonucleoprotein (RNP)^49^. YBX1 directly binds tiRNAs in a sequence-specific manner via its CSD and is required for packaging tiRNA-repressed mRNAs into SGs but not for tiRNA-mediated eIF4F displacement and translation inhibition.

The Polacek group reported another mechanism by which tsRNA inhibits global translation, where 26-nt ValGAC5′tsRNA of *Haloferax volcanii* inhibits in vitro translation by binding to the small ribosomal subunit and interfering with peptide bond formation^50^. Thus, the valine tsRNA is presented as a ribosome-binding small ncRNA capable of regulating gene expression in *H. volcanii* under environmental stress conditions to fine-tune the rate of protein synthesis. They further determined that the 26-nt ValGAC5′tsRNA could compete with mRNAs to bind to the small ribosomal subunit and inhibit global translation^51^.

The Bąkowska-Żywicka group also showed that tsRNAs regulate mRNA translation in *Saccharomyces cerevisiae*, which lacks miRNA machinery^52^. Five 3′tsRNAs and one 5′tsRNA were identified to interact with small ribosomal subunits and aminoacyl-tRNA synthetases (aa-RSs) and impair the efficiency of tRNA aminoacylation, leading to translation inhibition.

The Tavazoie group revealed that tRNA^TyrGUA^ depletion under oxidative stress inhibits the growth of normal-like mammary epithelial MCF10A cells by reducing the expression of growth-promoting genes enriched in tyrosine codons. In addition, augmented TyrGUA tRNA halves are produced in a tumor suppressor DIS3-like 3′-5′ exoribonuclease 2 (DIS3L2)-dependent manner and interact with the RNA-binding proteins heterogeneous nuclear ribonucleoprotein A1 (hnRNPA1) and small RNA-binding exonuclease protection factor La (SSB) to regulate their activities^53^.

In disagreement with other groups arguing that tRNA halves are responsible for SG formation, the Schaefer group proposed that tRNA fragmentation and SG formation are not concurrent events, reporting that SG formation is induced by treatment with inorganic sodium arsenite, NaAsO_2_ (As[III]), at concentrations that do not cause tRNA fragmentation. These observations suggest that tsRNA is produced after cells respond to As[III] via SG formation. Furthermore, it has been observed that the generation of tRNA fragments by oxidative stress increases cell death during the stress recovery phase^54^.

#### Translational activation

Another pathway in which tsRNAs are involved in translation activation, where 19-nt Gln5′tsRNA stimulates the translation of ribosomal and RNA-binding proteins. The mammalian multisynthetase complex (MSC) is the major binding partner of 19-nt Gln5′tsRNA and enhances ribosomal protein synthesis^55^.

The Kay group revealed that LeuCAG3′tsRNA binds to the mRNAs of ribosomal proteins RPS28 and RPS15 through Watson-Crick base-pairing, which alters the secondary structures of both mRNAs and enhances their translation^32^. Since RPS28 is a component of the 40S ribosome and is essential for 18S rRNA biogenesis, inhibition of the LeuCAG tsRNA blocks pre-18S ribosomal RNA processing and reduces the number of 40S ribosomes, leading to apoptosis^32,56^. LeuCAG3′tsRNA levels in mouse hepatocellular carcinoma are also higher than those in normal liver. Inhibition of LeuCAG3′tsRNA reduces tumor growth and induces apoptosis in an orthotopic patient-derived hepatocellular carcinoma (HCC) xenograft (PDX) model. These findings suggest that this tsRNA can be a potential target for HCC treatment. Furthermore, the target site of LeuCAG3′tsRNA in the RPS28 coding sequence is conserved in vertebrates, and LeuCAG3′tsRNA regulates RPS28 mRNA translation at the postinitiation stage in humans and mice. Therefore, LeuCAG3′tsRNA-mediated maintenance of ribosomal biogenesis might be a conserved process in vertebrates^57^ (Fig. 3b).

Unlike other eukaryotes, *Trypanosoma brucei* (*T. brucei*) mainly regulates gene expression at the post-transcriptional level since it lacks the ability to regulate the transcription of protein-coding genes. The Polacek group reported tsRNA-mediated translational activation in *T. brucei*, where a tRNA^Thr^ 3′half is produced during starvation or the stationary phase in procyclic cells and binds to ribosomes and polysomes to stimulate protein biosynthesis during the stress recovery phase^58^ (Fig. 3c).

### RNA-binding protein-associated regulation

#### Suppression: tsRNAs act as decoys

**Fig. 4:**
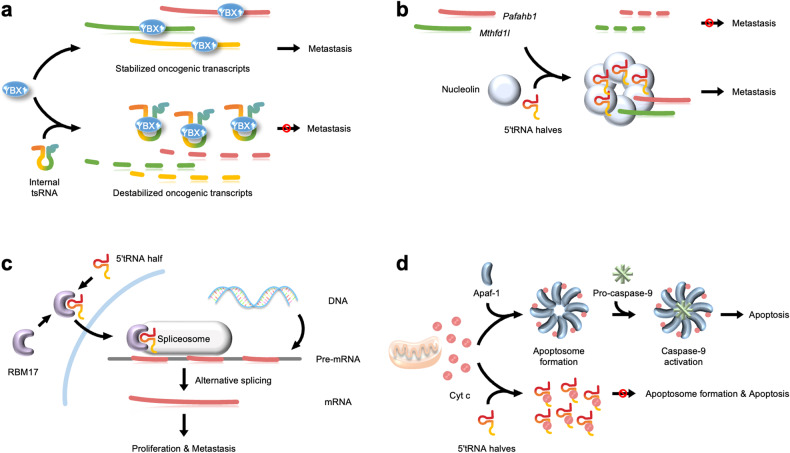
RNA-binding protein-associated regulation. **a** Suppression: tsRNAs act as decoys. Internal tsRNA (i-tRF) binds to Y-box binding protein 1 (YB-1 or YBX1) and interferes with the interaction of YBX1 with several oncogenic transcripts. This reduces the stability of oncogenic transcripts and suppresses the metastasis of breast cancer cells. **b** Stabilization: tsRNAs induce nucleolin oligomerization. The CysGCA5′tRNA half binds to nucleolin and enhances the interaction of nucleolin with platelet-activating factor acetylhydrolase 1 beta 1 (*Pafah1b1*) and methylenehydrofolate dehydrogenase 1 like (*Mthfd1l*) transcripts. This induces nucleolin oligomerization, which protects these transcripts from exonucleolytic degradation and increases their stability, thereby increasing metastasis. **c** Stabilization: tsRNAs induce protein translocation. The 33-nt-GlyGCC 5′tRNA half binds to RBM17 and promotes its translocation to the nucleus. Stabilized RBM17 in the nucleus induces alternative splicing of MAP4K4 mRNA, increasing papillary thyroid cancer (PTC) cell proliferation and metastasis. **d** Inhibition of apoptosis: tsRNAs act as competitors. The binding of cytochrome c (cyt c) to the caspase activator Apaf-1 induces apoptosis. The 5′tRNA half binds to cyt c and interferes with the interaction between cyt c and Apaf-1. This prevents apoptosome formation and inhibits apoptosis.

The Tavazoie group delineated that tsRNAs could regulate the levels of oncogenic transcripts associated with breast cancer metastasis through two different pathways^24,59^ (Fig. 4). Hypoxic stress-induced internal tsRNAs (i-tRF) inhibit the progression of breast cancer metastasis by binding to the oncogenic RNA-binding protein YBX1^24^. YBX1 is involved in key cellular pathways and is highly overexpressed in several cancer types^60–63^. Internal tsRNAs derived from tRNA^Glu^, tRNA^Asp^, tRNA^Gly^, and tRNA^Tyr^ bind to the YBX1 protein, thereby preventing the binding of several oncogenic transcripts to YBX1 (Fig. 4a). This reduces the stability of oncogenic transcripts in breast cancer cells and, as a result, suppresses breast cancer cell growth. The expression levels of i-tRFs are significantly lower in highly metastatic breast cancer cells than in breast cancer cells with low malignant potential, suggesting that highly metastatic cancers may have a mechanism to evade tsRNA-mediated suppression^24^.

#### Stabilization: tsRNAs induce nucleolin oligomerization

The Tavazoie group also determined that the CysGCA5′tRNA half regulates breast cancer progression by an additional mechanism that is independent of the i-tRFs mentioned in the previous section^59^ (Fig. 4b). The CysGCA5′tRNA half is upregulated during breast cancer progression and metastasis. It directly binds to nucleolin, an RNA-binding protein involved in various RNA processes^64–69^, and enhances the interactions between nucleolin and platelet-activating factor acetylhydrolase 1 beta 1 (*Pafah1b1*) and methylenetetrahydrofolate dehydrogenase 1 like (*Mthfd1l*) transcripts. It induces nucleolin oligomerization, which protects these transcripts from exonucleolytic degradation and increases their stability, leading to efficient metastatic lung colonization and survival of breast cancer cells^59^ (Fig. 4b).

#### Stabilization: tsRNAs induce protein translocation

The Liao group reported a new mechanism of tsRNA-regulated gene expression, in which tsRNA stabilizes the target protein with which it interacts. Papillary thyroid cancer (PTC) samples exhibit elevated levels of a 33-nt-GlyGCC 5′tRNA half, which binds to the UHM domain of RBM17 and promotes the translocation of RBM17 to the nucleus. Nuclear localization of RBM17 increases its stability by preventing its ubiquitin/proteasome-dependent degradation^70^. Stabilized RBM17 induces alternative splicing of exon 16 of *MAP4K4* mRNA, which leads to the proliferation and migration of PTC cells via the MAPK signaling pathway (Fig. 4c).

#### Inhibition of apoptosis: tsRNAs act as competitors

Apoptosis is a form of programmed cell death for the elimination of unneeded or abnormal cells in multicellular organisms. When apoptosis is not triggered properly, cells can grow and divide uncontrollably, leading to the formation of cancers. Loss of apoptotic control causes cancer cells to survive longer, increases invasiveness, and stimulates angiogenesis during cancer progression. One of the important roles of tRNA halves is the regulation of apoptosis in cancer cells.

The Hatzoglou group revealed that angiogenin-induced tRNA halves inhibit apoptosis to increase cell survival in mouse embryonic fibroblasts under hyperosmotic stress^71^. Apoptosis occurs when cytochrome *c* (cyt c) binds to caspase activator Apaf-1^72,73^. Approximately 20 tRNA halves are abundant in the cyt c-ribonucleoprotein (RNP) complex, and they block the interaction between Apaf-1 and cyt c^71^. Their presence prevents Apaf-1 oligomerization and apoptosome formation, resulting in the inhibition of apoptosis (Fig. 4d). In addition, primary cortical neurons are also protected from apoptosis by angiogenin under hyperosmotic stress^71,74^. These studies have revealed the association between angiogenin-induced tRNA halves and cell survival in response to hyperosmotic stress and proposed a novel cellular complex inhibiting apoptosis. In addition, cancer cells may utilize this strategy for apoptosis resistance. Thus, angiogenin-induced tRNA halves may be an important target for cancer therapy.

Breast cancer tissues exhibit elevated levels of tsRNA-26576, which inhibits cellular apoptosis and induces cellular multiplication and migration in MDA-MB-231 cells^75^. Tumor suppressor genes, including FAT4 and SPEN, are also upregulated by tsRNA-26576 inhibition in MDA-MB-231 cells. Therefore, tsRNA-26576 upregulation in breast cancer may inhibit apoptosis while promoting cell growth; thus, tsRNA-26576 may serve as a therapeutic target and predictive marker for breast cancer. In addition, 75 tsRNAs were upregulated and 188 were downregulated in breast cancer tissues compared with adjacent normal tissues. These results suggest that various tsRNAs are involved in breast cancer and can be targeted for cancer therapy.

Another tsRNA, 3′tsRNA-ValCAC-2, directly binds to the chaperone molecule EEF1A1 (which has oncogenic functions in various cancers) and mediates the transport of EEF1A1 to the nucleus, which promotes its interaction with MDM2, a p53 E3 ubiquitin ligase. This leads to gastric cancer progression and suppression of apoptosis through inhibition of the MDM2-p53 pathway^76^.

### RNA modifications

There are more than 100 types of RNA nucleotide modifications. tRNAs are heavily modified and have an average of 11–14 modifications, which are added during tRNA maturation. Since these modifications affect the stability and function of various types of RNA, a number of methods to detect RNA modifications have been developed^77^. These RNA modifications also regulate tsRNA stability, function, and biogenesis. The effects of several modifications on tsRNAs have been investigated in relation to neurological abnormalities. Currently, an increasing amount of evidence also suggests that RNA modifications can be crucial for tsRNA function in tumorigenesis. Here, we will summarize the roles of tsRNA modifications and the enzymes catalyzing them (Fig. 1).

#### DNMT2

In 2010, the Lyko group revealed that *Drosophila* DNA methyltransferase Dnmt2-mediated cytosine methylation at C38 in the anticodon loop of cytoplasmic tRNA^AspGTC^, tRNA^ValAAC^, and tRNA^GlyGCC^ protected them from angiogenin-mediated tRNA cleavage^78^. The Chen group also revealed that DNMT2-mediated modification regulates tsRNA expression. Deletion of mouse DNMT2 blocks RNA modification (m^5^C and m^2^G) in 30–40-nt sperm RNA fractions and alters the small RNA expression profile of sperm, including tsRNAs, eliminating the transmission of high-fat diet (HFD)-induced metabolic disorders^79^. The Zhou group discovered that 30–40-nt tsRNAs are required for the transmission of HFD-induced paternal metabolic disorders to offspring. m^5^C and m^2^G are increased in 30–40-nt sperm tsRNAs^80^. HFD can upregulate Dnmt2 in the caput epididymis, consistent with an increase in m^5^C in sperm 30–40-nt RNA fractions. Dnmt2 deletion decreases the levels of rRNA-derived small RNAs, induces m^5^C hypomethylation, promotes tRNA fragmentation, and alters the chemical and biological properties of tsRNAs^79^.

#### NSUN2

NSUN2 is a cytosine-5 RNA methyltransferase responsible for up to 60–80% of 5-methylcytosines (m^5^C) at positions 48, 49, and 50 of tRNA molecules in both human and mouse cell lines^81^. It is the most widely investigated tRNA modification enzyme thus far. The Lyko group reported that a lack of either Dnmt2 or cytosine-5 methylase Nsun2 does not affect mouse viability. However, double mutants exhibit an underdeveloped phenotype and impaired cellular differentiation. Loss of Dnmt2 and Nsun2 decreases the rate of protein synthesis and induces tRNA degradation. These observations establish an important function of cytosine-5 tRNA methylation in mouse development^82^.

The Kuss group observed severe short-term memory (STM) loss upon deletion of CG6133, the *Drosophila* ortholog of Nsun2. Moreover, individuals with Nsun2 mutations show both intellectual disability and facial dysmorphism phenotypes. NSUN2 is required for nucleotide modification at the wobble position of the tRNA^LeuCAA^ anticodon. Therefore, the loss of NSUN2 function can lead to an absence of tRNA^LeuCAA^ and consequently change tissue-specific protein expression by altering codon usage, affecting the development of the fetal brain. Thus, NSUN2 may play a role in the translational regulation required for proper synaptic plasticity, learning, and memory^83^.

The Frye group has also confirmed that loss of NSUN2-mediated m^5^C in tRNA contributes to neurodevelopmental disease. Angiogenin cleaves unmethylated tRNA rather than methylated tRNA. Accumulation of 5′tRNA halves through stress-induced angiogenin-mediated tRNA cleavage in the absence of NSUN2 reduces protein translation rates and activates stress pathways, resulting in reduced cell size and increased apoptosis in cortical, hippocampal, and striatal neurons. Thus, mutations in Nsun2 cause microcephaly and other neurological abnormalities in mice and humans^84^. It has been further confirmed that both the presence of angiogenin and the depletion of NSUN2 inhibit neural cell migration toward chemoattractant fibroblast growth factor 2 and suppress the neural differentiation of neuroepithelial stem cells, suggesting that NSUN2-mediated tRNA methylation is required for efficient migration and differentiation of intermediate progenitors for upper-layer neurons^85^.

NSUN2-mediated m^5^C regulates the cellular metabolic state and global protein synthesis in response to external stress stimuli by acting as a metabolic sensor for external stress. Oxidative stress inhibits NSUN2 and decreases m^5^C levels at specific tRNA sites, which alters tsRNA biogenesis and impairs protein synthesis. Deletion of NSUN2 also affects three major metabolic pathways: the methionine cycle, amino acid synthetic pathways, and the tricarboxylic acid (TCA) cycle. These events result in increased protein degradation, inhibition of protein synthesis, and enhanced glycolysis. Taken together, these observations suggest that site-specific methylation of tRNAs changes dynamically during cellular stress conditions even within the same tRNA molecule, which regulates the biogenesis of tsRNAs^86^.

The Schaefer group has shown another role of m^5^C in tRNA; mutations in *Dnmt2* and *Nsun2* reduce tRNA stability and increase the levels of tsRNAs, suggesting that m^5^C impairs genome integrity including LTR-containing TEs and telomere^87^

#### PUS7

The Bellodi group has revealed that the stem cell-enriched pseudouridine (Ψ) “writer” PUS7 is another regulator of global translation^88^. PUS7 synthesizes Ψ at the 8^th^ nucleotide of 18-nt TOG-containing 5′tsRNAs. Ψ-bearing 5′tsRNA interferes with the binding of translation initiation factor, eIF4G and polyadenylate-binding protein cytoplasmic 1 (PABPC1). Therefore, this event impairs tsRNA-mediated global translation, which affects early embryogenesis and hematopoietic commitment. These findings suggest that disruption of Ψ-driven regulatory circuits may contribute to human myeloid malignancy^88^.

#### TRMT2A

The tRNA methyltransferase TRMT2A catalyzes the 5-methyluridine (m^5^U) modification at position 54 of cytosolic tRNAs. The Soares group found that TRMT2A knockdown induces m^5^U54 tRNA hypomodification and leads to an increase in tsRNA levels. Hypomodification of m^5^U54 leads to angiogenin upregulation to generate 5′tiRNAs and affects cellular stress responses and RNA stability. Under oxidative stress, TRMT2A is downregulated, and tiRNA formation is promoted. Thus, m^5^U54 can be a protective modification against tRNA cleavage^89^.

#### TRMT6/61A

Unlike TRMT2A, which regulates the biogenesis of tsRNAs, TRMT6/61 A is involved in tsRNA function. TRMT6/61A-dependent m^1^A is enriched in 22-nt 3′tsRNAs (tRF-3bs). The Dutta group identified that TRMT6/61A-dependent m^1^A causes problems in the assembly of tRF-3b and its target mRNAs. However, it does not affect the interaction between tsRNA and AGO2. Elevated levels of m^1^A modification have been detected in urothelial carcinoma of the bladder upon TRMT6/61 A overexpression and are associated with dysregulation of the tRF targetome. TRMT6/61A-mediated tRF-3b modification plays a role in the maintenance of the unfolded protein response (UPR). Since rapidly proliferating cancer cells maintain protein homeostasis by activating the pro-survival UPR to relieve ER stress, the UPR may be a potential target for cancer therapy^90^.

## Potential biomarkers and therapeutic targets in cancers

**Fig. 5:**
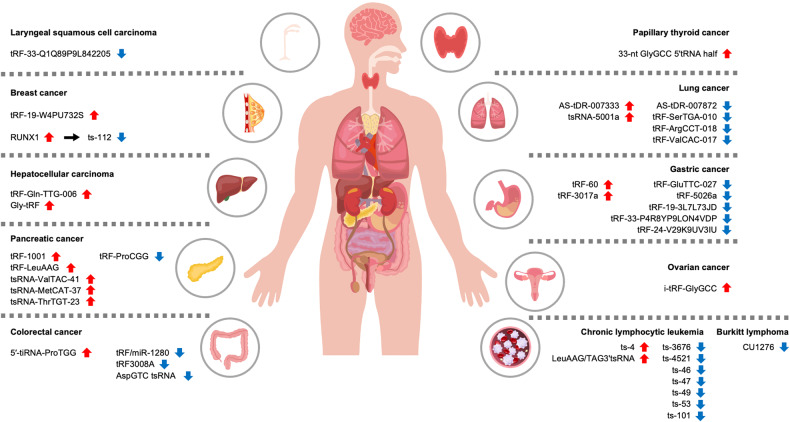
Potential biomarkers and therapeutic targets in cancers. The characteristic expression of tsRNAs in various cancers. A blue downward arrow shows a decrease in the indicated tsRNA, and a red upward arrow shows an increase in the indicated tsRNA.

To date, a number of studies have revealed that tsRNAs play important roles in the development and progression of various cancers by acting as promoters or suppressors of cancer. Although the underlying mechanism of tsRNA function in tumorigenesis needs to be further determined, many tsRNAs show characteristic expression patterns and are linked to disease prognosis in cancer. For example, tRF-20-S998LO9D (ArgTCT5′tsRNA) is highly expressed in various cancers and is associated with phenotypes of poor prognosis, such as increased cell proliferation, so it is thought to have an oncogenic function^91^. In the next section, we will delineate a number of tsRNAs that were not introduced in the previous section according to specific cancer types (Fig. 5).

### Chronic lymphocytic leukemia (CLL)

ts-3676, ts-4521, ts-46 (HisGTG type II tsRNA), ts-47 (ArgTCG type II tsRNA), ts-49 (ThrCGT type II tsRNA), ts-53 (ThrAGT type II tsRNA), and ts-101 (SerGCT type II tsRNA) are downregulated, while ts-4 (PseudoCTT type II tsRNA) is upregulated in CLL and lung cancer^21,22^. In addition, it has been revealed that ts-46 and ts-47 can act as tumor suppressors through a colony assay^22^.

B-cell chronic lymphocytic leukemia (B-CLL) exhibits upregulated levels of LeuAAG/TAG3′tsRNA. The targets of LeuAAG/TAG3′tsRNA are predicted to be related to cell cycle regulation, metabolism pathways, ribosome biogenesis, and signaling pathways such as PI3K/AKT, Wnt, and NFκB. High levels of LeuAAG/TAG3′tsRNA are correlated with inferior overall survival (OS)^92^.

### Breast cancer (BC)

In BC, tRF-19-W4PU732S (19-nt SerAGA5′tsRNA) is upregulated and induces cell proliferation, migration, invasion, epithelial-to-mesenchymal transition (EMT), and cancer stem-like cell (CSC) phenotypes by targeting RPL27A, a member of the large ribosomal proteins through an unknown mechanism^93^.

In addition, when the expression of the tumor suppressor runt-related transcription factor 1 (RUNX1), a key factor in the onset and metastasis of BC, is suppressed in normal-like mammary epithelial MCF10A cells, ts-19 (32-nt ValCAC type II tsRNA) and ts-29 (24-nt TyrGTA type II tsRNA) are significantly downregulated, while ts-46 (33-nt HisGTG type II tsRNA) is upregulated. When RUNX1 is overexpressed, ts-112 (27-nt SerGCT type II tsRNA) is downregulated, which prevents excessive cell proliferation in MCF10A and BC cell lines. Increased levels of a ts-112 mimic in MCF10A cells promote cell proliferation, suggesting that ts-112 might have oncogenic activity^23^.

### Lung cancer

In non-small cell lung carcinoma (NSCLC), AS-tDR-007333 (28-nt GlyGCC 5′tsRNA) levels are elevated, whereas AS-tDR-007872 (derived from ArgCCT) levels are reduced^94,95^.

AS-tDR-007333 enhances the malignancy of NSCLC cells by activating MED29 through two different mechanisms. In the first mechanism, AS-tDR-007333 directly interacts with HSPB1 (HSP27) to increase MED29 expression by altering histone modifications in the promoter of MED29. In the second mechanism, AS-tDR-007333 increases the expression of ELK4, which binds to the MED29 promoter and promotes its transcription. Therefore, AS-tDR-007333 is considered a potential diagnostic or prognostic marker and therapeutic target for NSCLC^95^.

AS-tDR-007872 acts as an antioncogene in pulmonary adenocarcinoma (PADC). It regulates apoptosis by suppressing the expression of BCL2L11 and is considered a potential biomarker for lung cancer^94^.

In lung adenocarcinoma tissues, tsRNA-5001a (15-nt TyrGTA5′tsRNA) is upregulated, while tRF-SerTGA-010 (22-nt SerTGA type II tsRNA), tRF-ArgCCT-018 (32-nt ArgCCT type II tsRNA), and tRF-ValCAC-017 (16-nt ValCAC type II tsRNA) are downregulated^96,97^. Among them, tsRNA-5001a is associated with a poor prognosis and an increased risk of recurrence after surgery. It is speculated that tsRNA-5001a promotes tumor growth and worsens the prognosis of lung cancer by binding to the antitumor gene GADD45G (Growth Arrest and DNA Damage Inducible Gamma) and downregulating its expression^96^.

Target genes of tRF-SerTGA-010, tRF-ArgCCT-018, and tRF-ValCAC-017 are predicted to be related to the MAPK signaling pathway based on KEGG pathway analysis. They are also anticipated to be related to nervous system development, Golgi transport complex, and endodeoxyribonuclease cleavage activity based on GO pathway analysis^97^.

### Gastric cancer (GC)

In GC, tRF-60 (17-nt ValCAC3′tsRNA) and tRF-3017a (19-nt ValTAC3′tsRNA) are upregulated^76,98^. In contrast, tRF-GluTTC-027 (17-nt GlyTTC type II tsRNA), tRF-5026a (tRF-18-79MP9P04 or 18-nt ValAAC5′tsRNA), tRF-19-3L7L73JD (19-nt ValAAC i-tRF), tRF-33-P4R8YP9LON4VDP (33-nt GlyGCC 5′tRNA half), and tRF-24-V29K9UV3IU (24-nt mitochondrial GlnTTG5′tsRNA) are downregulated^99–103^.

GC progression is inhibited by tRF-GluTTC-027, which inhibits the MAPK signaling pathway that affects the oncologic characteristics of GC cell lines^102^. Another tsRNA, tRF-5026a, regulates the PTEN/PI3K/Akt signaling pathway, thereby inhibiting GC cell growth and acting as a tumor suppressor^103^. The expression of tRF-19-3L7L73JD is associated with tumor size and inhibits cell proliferation and migration. It also induces apoptosis and arrests the cell cycle at the G0/G1 phase^99^. The proliferation and migration of BGC-823 and SGC-7901 cell lines are inhibited by tRF-33-P4R8YP9LON4VDP^100^. The tsRNA tRF-24-V29K9UV3IU inhibits proliferation, invasion, and metastasis by silencing G protein-coupled receptor 78 (GPR78), a central node of various signal transduction pathways^101^. Finally, tRF-3017A interacts with AGO2 to suppress nerve epidermal growth factor-like like protein 2 (NELL2) and promotes GC cell migration and invasion^98^.

### Burkitt lymphoma

In Burkitt’s lymphoma, CU1276 (22-nt GlyGCC3′tsRNA) is downregulated and is known to regulate cell proliferation and DNA repair by targeting RPA1, which has well-characterized roles in DNA replication and repair^11^.

### Hepatocellular carcinoma (HCC)

tRF-Gln-TTG-006 (22-nt GlnTTG5′tsRNA) and Gly-tRF (30-nt GlyGCC 5′tsRNA) are upregulated in serum and tissues of HCC, respectively^104,105^. The latter inhibits NDFIP2 and activates the AKT signaling pathway, which enhances liver cancer stem cell (LCSC)-like properties and promotes HCC migration^105^.

### Laryngeal squamous cell carcinoma (LSCC)

In LSCC, tRF-33-Q1Q89P9L842205 (GlyCCC 5′tRNA half) is downregulated, which is linked to lymph node metastasis and advanced stages of LSCC. It represses the phosphoinositide 3-kinase catalytic subunit (PIK3CD), which promotes apoptosis and inhibits cell migration, invasion, and proliferation^106^.

### Papillary thyroid cancer

In papillary thyroid cancer, the 33-nt GlyGCC 5′tRNA half is upregulated and directly binds to RBM17 protein to induce alternative splicing of MAP4K4 mRNA, thereby showing tumor-promoting effects^70^.

### Ovarian cancer

Epithelial ovarian cancer (EOC), which shows frequent recurrence, chemotherapy resistance, and poor 5-year survival, exhibits elevated levels of i-tRF-GlyGCC. Increased i-tRF-GlyGCC levels are associated with an aggressive ovarian tumor phenotype, early progression, and adverse survival outcomes after debulking surgery and platinum-based chemotherapy^107^.

### Pancreatic cancer (PC)

In PC, tRF-1001 (SerTGA type II tsRNA), tRF-LeuAAG (LeuAAG3′tsRNA), tsRNA-ValTAC-41 (41-nt ValTAC3′tsRNA), tsRNA-MetCAT-37 (37-nt MetCAT3′tsRNA), and tsRNA-ThrTGT-23 (23-nt ThrTGT3′tsRNA) are upregulated, whereas tRF-ProCGG (tRF-001391, ProCGG 5′tRNA half) is downregulated^5,108–110^. Among these, tRF-1001 is involved in cell proliferation by regulating the cell cycle^5^, and tRF-LeuAAG promotes cell proliferation and migration by downregulating upstream frameshift mutant 1 (UPF1) expression^108^. Patients with low serum levels of tsRNA-ValTAC-41 have longer survival times, and tsRNA-ValTAC-41 level is also positively correlated with the AJCC stage of pancreatic ductal adenocarcinoma (PDAC)^110^. Low expression of tRF-ProCGG is associated with poor survival and poor prognosis in PDAC patients^109^.

### Colorectal cancer (CRC)

In CRC, 5′tiRNA-ProTGG is upregulated, whereas tRF/miR-1280 (17-nt LeuAAG/TAG3′tsRNA), tRF3008A (17-nt ValAAC/CAC3′tsRNA, AS-tDR-000076), and AspGTC tsRNA (tRF-19-R959KU1Y) are downregulated. These changes are significantly associated with metastatic stage^111–113^. Among these, tRF/miR-1280 is an important regulator of cancer stem cell (CSC) growth and function in CRC by the regulation of Notch/Gata and miR-200b signaling^112^. High levels of ProTGG5′tiRNA are correlated with poor disease-free survival (DFS) and poor OS in CRC patients. Predicted target genes of ProTGG5′tiRNA are related to cellular communication and signaling, various developmental processes, and cellular motility^111^. The tsRNA tRF3008A suppresses CRC proliferation and EMT by suppressing Wnt/β-catenin signaling through inhibition of FOXK1 in an AGO-dependent manner^113^.

### Extracellular tsRNAs

RNA-containing vesicles and membrane-free RNA/protein complexes can be transported from cells to the extracellular environment, affecting the functioning of target cells. Extracellular RNA contains sequences derived from a number of different species of RNA, including a subset of tsRNAs^114^.

The Ansel group found that 45% of tsRNAs and less than 1% of miRNAs are present in extracellular vehicles (EVs) compared to primary T cells. tsRNAs are selectively secreted from T cells through multivesicular bodies (MVBs), suggesting that this process might help eliminate tsRNAs causing immunosuppression^115^. The Martin group has elucidated that there are abundant 5′tRNA halves derived from tRNA^Gly^, tRNA^Val^, tRNA^Glu^, tRNA^His^, and so on in mouse serum. These 5′tRNA halves are present as macromolecular complexes of size 100–300 kDa in blood. Additionally, the amount of 5′tRNA halves in serum changes with age and in response to caloric restriction. Their characteristic regulation patterns and known functions, i.e., global translation inhibition, suggest that 5′tRNA halves may constitute a new type of signaling molecule^116^. The Peng group also demonstrated that tsRNAs are ubiquitously present in exosomes and are drastically elevated in plasma exosomes of liver cancer patients compared with healthy donors, suggesting that tsRNAs may be used as biomarkers for cancer diagnosis^117^.

## Closing remarks

To date, approximately 200,000 papers have been published on a variety of ncRNAs, including tsRNAs. The approach used for miRNA research has often been applied to the investigation of other types of small ncRNAs with less rigorous experimental design^118^. As the length of tsRNAs is similar to that of miRNAs and their sequence contains a portion of full-length tRNAs, carefully designed experiments and the critical interpretation of the data is necessary to distinguish tsRNAs from miRNAs or tRNAs before the conclusions of the tsRNA papers can be accepted. Such prudent efforts will lead to a better and more reliable understanding of tsRNA biology and roles, contributing to the development of applications including gene therapy.

Recent developments in the field of human gene therapy have raised the prospect of engineering therapeutics based on short RNAs such as tsRNAs, especially for cancer treatment. Building on these observations, various therapeutic strategies can be envisioned to target or restore specific tsRNA species and other small RNAs. Antisense oligonucleotides (ASOs) have been approved or are in late-stage development for spinal muscular atrophy, familial hypercholesterolemia, transthyretin-mediated amyloidosis, and forms of Duchenne muscular dystrophy, among many other conditions^119–121^. Delivery of ASOs or other chemically modified oligonucleotides might augment or inhibit the expression of targeted tsRNA species as well as full-length tRNAs. Viral vectors such as adeno-associated viruses (AAVs) can also be harnessed to express short RNA molecules or to deliver several copies of a complementary sequence to sponge aberrantly expressed tsRNAs. Another potential strategy would be the targeting of proteins that add RNA modifications that affect tsRNA biogenesis and function. In addition, tsRNA profiles can be used as biomarkers in the context of clinical trials, for example, in gastric cancer^122^. A better understanding of the regulatory relationships between tsRNAs and their host tRNA molecules could also lead to novel therapeutic strategies; for example, several companies have dedicated resources for tRNA therapeutics, including codon optimization and delivery of suppressor tRNAs to read through premature stop codons^123^. As we better understand the causal roles of individual tsRNAs in cancer development, all these strategies can readily be adopted for therapeutic purposes.
